# Advanced Technologies for Studying Microbiome–Female Reproductive Tract Interactions: Organoids, Organoids-on-a-Chip, and Beyond

**DOI:** 10.1055/s-0043-1778067

**Published:** 2024-01-23

**Authors:** Yosun A. Kaya, Marcel R. de Zoete, Gaby S. Steba

**Affiliations:** 1 Division of Female and Baby, Department of Reproductive Medicine and Gynaecology, University Medical Centre Utrecht, Utrecht, The Netherlands; 2 Department of Medical Microbiology, University Medical Centre, Utrecht, The Netherlands

**Keywords:** female reproductive tract, organoids, in vitro models, microbiome, host–microbe interactions

## Abstract

The female reproductive tract (FRT) is home to diverse microbial communities that play a pivotal role in reproductive health and disorders such as infertility, endometriosis, and cervical cancer. To understand the complex host–microbiota interactions within the FRT, models that authentically replicate the FRT's environment, including the interplay between the microbiota, mucus layer, immune system, and hormonal cycle, are key. Recent strides in organoid and microfluidic technologies are propelling research in this domain, offering insights into FRT–microbiota interactions and potential therapeutic avenues. This review delves into the current state of FRT organoid models and microbe integration techniques, evaluating their merits and challenges for specific research objectives. Emphasis is placed on innovative approaches and applications, including integrating organoids with microfluidics, and using patient-derived biobanks, as this offers potential for deeper mechanistic insights and personalized therapeutic strategies. Modeling various FRT properties in organoids is explored, from encompassing age-related epithelial features, oxygen levels, and hormonal effects to mucus layers, immune responses, and microbial interactions, highlighting their potential to transform reproductive health research and predict possible outcomes.

We are only beginning to unravel the complex interplay between the human body and its resident microorganisms, collectively known as the microbiota, which will shed light on the role of microorganisms in various aspects of human health, including nutrition, immunity, and disease susceptibility. 1 Within this paradigm, the female reproductive tract (FRT) emerges as a critical arena where microbial interactions profoundly influence reproductive health and fertility. The FRT is composed of different anatomical regions, each with its unique physiological and microbial communities that play a significant role in maintaining reproductive health and fertility. 2 For example, the microbiome of the fallopian ampulla tube significantly differs from the isthmus. 3 However, it was shown that ∼70% of the microbial communities of the endometrium and the fallopian tubes (FTs) are shared within a patient. 4 It is acknowledged that the microbiome plays a role in the process of becoming pregnant and maintaining a healthy pregnancy. 5 Consequently, alterations in microbial homeostasis, sometimes referred to as dysbiosis, can lead to various reproductive disorders, ranging from bacterial vaginosis and pelvic inflammatory disease (PID) to infertility and pregnancy complications. 6 7 8 Yet, there remains a lot to be explored regarding this subject as knowledge of how the microbiome influences reproduction remains inconclusive. This is partly due to the lack of comparability of studies on the microbiome of the FRT, regarding sampling methods, study set-up, and analysis. To address this, there is a need for well-designed clinical studies complemented by comprehensive metadata that can facilitate a proper analysis of the FRT microbiota's impact on reproductive health and disease. 9 To date, many studies have been published on the microbial composition of the FRT, though only a few functional studies have been performed on the interaction between the FRT and their microbiota. 10 11 12 13 14 15 There is thus a need for in vitro models to study the interactions between the FRT and the microbiota and its metabolites to better understand how the microbiota influences the female reproductive organs and to move the field from correlation to causation.

Organoid models have emerged as a powerful tool for studying host–microbe interactions in the FRT. 16 Organoids are three-dimensional (3D) cell culture systems that mimic the structure and function of the tissue of origin, providing a more physiologically relevant environment for studying these complex interactions. 17 In this review, the diversity of the FRT microbiome will be highlighted after which the current state of the technologies for culturing FRT organoids together with bacteria will be discussed. We will explore the progression of organoid (derived) models used to study host–microbe interactions, beginning with fundamental 3D organoid structures and their two-dimensional (2D) equivalents, then moving on to the intricate microfluidic-based systems. Which of these models have the potential to mimic the physical and mechanical properties of the FRT will be discussed, enhancing in vitro study capabilities. The ideal system ought to be able to model the complex interplay between mucus, mucins, and host–microbe interactions within the FRT, as this is essential for reproductive health and immunity. 18 However, it will depend on the researchers' goals which model will suit best. The review provides an overview of the available models and discusses what features can be integrated to create more representative in vitro models.

The lower reproductive tract is the most well-studied part of the FRT. The cervix is dominated by *Lactobacillus* species such as *Lactobacillus crispatus* , *Lactobacillus gasseri* , *Lactobacillus jensenii* , and *Lactobacillus iners* . These lactic acid-producing bacteria, in particular *L. crispatus* , promote a low pH environment (pH < 4.5), which is unfavorable for the growth of unwanted bacteria. 19 Dysbiosis can lead to conditions such as cervicitis. 2 20 21 More importantly, studies have shown that cervical dysbiosis, characterized by reduced *Lactobacillus* and increased diversity of anaerobic bacteria, is associated with an increased risk of cervical intraepithelial neoplasia and cervical cancer. 22 Some studies also suggest a potential link between the cervical microbiome and human papillomavirus infection, the primary cause of cervical cancer. 23 It has been established that, like the cervix, the vagina is home to a microbial community that is predominantly composed of *Lactobacillus* species. Dysbiosis in the vaginal microbiota can lead to bacterial vaginosis, characterized by overgrowth of anaerobic bacteria like *Gardnerella vaginalis* , *Prevotella* spp., and *Mobiluncus* spp. 24 The presence of these unwanted bacteria is associated with an increased pH, causing a range of symptoms from unpleasant odors to itching and burning. 21 Studies have also linked vaginal dysbiosis to higher susceptibility to sexually transmitted infections, including human immunodeficiency virus 1 (HIV-1). 25

Traditionally, the upper reproductive tract, including the FTs and the endometrium, has been considered sterile. However, emerging studies have challenged this concept, identifying a unique and less diverse microbial community in the FTs compared with the lower reproductive tract predominantly consisting of *Pseudomonas* , *Staphylococcus* sp., and *Prevotella* species. 3 According to many studies, the endometrial microbiome primarily consists of *Lactobacillus* spp., supplemented by Bacteroidetes, Proteobacteria, and Actinobacteria phyla. 3 26 27 28 29 30 31 There is a lesser presence of *Gardnerella* , *Streptococcus* , and *Bifidobacterium* spp. 32 Interestingly, the *lactobacillus* dominance within the endometrium is challenged by studies that obtained endometrial biopsies from hysterectomy, 33 laparoscopy, 34 and/or during caesarean sections. 35

Even though there is no clear consensus on the FRT microbial composition and its role in health and disease, a *Lactobacillus* -rich endometrial microbiome is linked to a higher likelihood of live birth. 19 In contrast, the presence of bacteria, particularly from the *Gardnerella* or *Streptococcus* genera, was associated with implantation issues or early pregnancy termination. 36 Moreover, microbial dysbiosis in the FTs may be associated with diseases like hydrosalpinx and PID, which can lead to infertility and ectopic pregnancy. 3

## Established Organoid Models of the FRT

Established organoid models of the FRT include organoids of the endometrium, FT, ovaries, and cervix and have previously been summarized by Alzamil et al. 16 Endometrial organoids (EMOs) that form gland-like structures and respond to hormones enable the study of the menstrual cycle and infertility-related defects. 37 38 FT organoids, containing both secretory and ciliated cells, model oviduct physiology. 39 There are two main methods for FT organoids, the air–liquid interphase system and self-organizing organoids. 40 Cervical organoids effectively replicate both squamous and columnar epithelium, offering valuable insights into cervical biology. 41 42 These organoids, developed from both human and mouse tissues, rely on specific growth factor combinations ( Table 1 ) for their formation and long-term expansion, with distinct requirements for ecto- and endocervical organoids. 43 They serve as pivotal models for understanding cervical homeostasis, with their reactions to factors like Wnt growth mirroring in vivo dynamics, such as metaplasia at the squamocolumnar junction. Moreover, recent advancements have led to the creation of ovarian cancer organoids, which closely resemble the original tumors in histological and genomic features, offering potential for drug screening and understanding tumor dynamics. 43 44 Kopper et al demonstrated the development of organoids from human ovarian surface epithelium, especially from women predisposed to ovarian cancer due to BRCA1/2 mutations. 45 These ovarian surface epithelium organoids, mirroring features like FT organoids with KRT8+ markers and distinct folds, face growth and long-term maintenance challenges. On the other hand, murine ovarian surface epithelium organoids thrive and can be perpetually cultured under certain conditions. 45 These innovative models showcased ciliated and secretory cells, but optimization was needed to model ovulation. Recently, ovarian organoids, or “ovaroids,” containing oocyte progenitor cells and supporting granulosa cells have been generated from human-induced pluripotent stem cells (iPSCs). 46 These ovaroid cells model follicle development, oocyte maturation, and hormone secretion. Lastly, robust human vaginal tissue models are still in development. 43 Currently, mouse-derived organoids replicate the in vivo architecture of vaginal tissue, displaying a stratified squamous epithelium with TP63+ cells. The precise modulation of Wnt is crucial for vaginal epithelial behavior, making this model pivotal for exploring vaginal epithelium regeneration and stability. 47

**Table 1:** Culture conditions and niche factors used for female reproductive tract organoids

Organoid	Key niche factors
Ovarian	Noggin, RSPO1, WNT3A, FGF10, nicotinamide
Fallopian tube	WNT3A, RSPO1, EGF, FGF10, Noggin
Endometrial	WNT3A, RSPO1, EGF, FGF10, Noggin, and A83–01
Cervical	RSPO1, Noggin, EGF, and Jagged-1
Vaginal	Ultraserum-G, EGF, A83–01 (TGFβ/Alk inhibitor), and Y-27632 dihydrochloride (ROCK inhibitor)

The majority of FRT organoids are derived from adult stem cells located within epithelial tissue fragments isolated from surgical samples or biopsies. 16 The tissue is enzymatically digested, and the fragments containing stem cells are embedded in extracellular matrix gels and overlaid with defined media to promote the proliferation of stem cells and their self-organization into organoids. 39 48 The specific media formulations vary based on the source of the FRT tissue but typically contain niche factors like WNT activators like WNT3A, bone morphogenetic protein, transforming growth factor inhibitors like A83–01, and mitogens like epidermal growth factor (EGF), R-spondin 1 (RSPO1), and fibroblast growth factor 10 (FGF10) to support stem cell growth while preventing differentiation ( Table 1 ). 37

## Introducing Bacteria to Organoid Systems: Selecting the Best Technique

Choosing an accurate method for introducing microbes or microbial products to organoids is vital for effective in vitro modeling of host–microbe interactions within the FRT. 49 The type of microbes involved, compatibility with natural conditions like pH and oxygen levels, as well as study requirements such as duration, scalability, and throughput must be considered. While many studies focus primarily on bacterial abundance, it is important to mention that bacterial load, as for instance quantified by 16S qPCR, is believed to significantly contribute to the effects of dysbiosis on the FRT health, 50 which should be considered by choosing the right bacterial concentration when studying interactions in a co-culture. By evaluating these variables, researchers can select the organoid system that best aligns with their goals, ensuring consistency and a robust understanding of complex relationships within the FRT. Several options have been emerged, namely, suspension coculture, fragmentation of the organoids, micro-injection of the organoids, and organoid polarity reversal ( Fig. 1 ) which will be described later. Each model has its advantages and disadvantages of which an overview is given in Table 2 .

**Table 2:** Advantages and disadvantages of organoid models to study host–microbiota interactions in the female reproductive tract

Model	Advantages	Limitations	References
Suspension culture	*Simplicity* : Easy to set up and handle. *Scalability* : Suitable for large-scale cultivation. *Uniformity* : Can provide homogeneous cell distribution and exposure to nutrients. *Cost-effective* : Less expensive in terms of equipment and materials	*Limited complexity* : May not accurately mimic in vivo conditions or interactions. *Shear stress* : Cells can be damaged due to agitation. *Lack of structure* : Absence of tissue architecture may not allow for realistic host–microbe interactions	14 15 51
Fragmentation	*Preservation of structure* : Maintains some of the in vivo architecture. *Versatility* : Can be applied to various tissues. *Accessibility* : Does not require highly specialized equipment	*Inconsistency* : Fragment size and shape can be variable, affecting results. *Potential damage* : Risk of harming cells during the fragmentation process	11 12 51
Microinjection	*Precision* : Allows for targeted delivery of microbes or substances. *Control* : Enables control over the quantity and location of the injection. *Applicability* : Strict anaerobes can be introduced for shorter assays	*Technically challenging* : Requires specialized skills and equipment. *Time-consuming* : Not suitable for high-throughput studies (if done manually) *Risk of damage* : Potential harm to cells at the injection site. Cannot sample microbiota during co-culture	51 55 57 64 67
Polarity reversal	*Access to apical surface* : Reversing the polarity exposes the apical surface, providing a more realistic platform for studying host–microbe interactions. Can sample microbiota during co-culture. *Enhanced study of specific interactions* : Offers a novel perspective to explore specific cellular interactions, including those involving luminal microorganisms. *Integration with other models* : Can be combined with other in vitro methods, expanding the range of possible studies. *Potential for personalized medicine* : May allow for individualized analyses based on patient-derived organoids, leading to more tailored treatments	*Complex procedure* : Reversing polarity might require advanced techniques and expertise, making it more challenging to implement. *Potential loss of structural integrity* : The procedure might disrupt the integrity of the organoid, affecting the accuracy of the model. *Limited applicability* : May not be suitable for all types of organoids or tissues, restricting its universal use. Can only sustain the growth of facultative anaerobes for short-term assays. *Cost and resource intensive* : Might require specialized equipment and reagents, adding to the overall cost and complexity of the study. *Potential for artifacts* : Manipulating organoid polarity might induce artificial conditions that do not accurately reflect the in vivo situation, leading to potential misinterpretations of the results	55 67 66
2D monolayers	*Controlled environment* : Facilitates the study of specific interactions. *Accessibility* : Easier to image and manipulate compared with 3D models. *Standardization* : Allows for more consistent and replicable conditions	*Limited complexity* : Lack of 3D structure might not fully represent in vivo conditions. *Potential for artificial interactions* : Might not accurately mimic host–microbe relationships	70 71 72 73
Air–liquid interfaces	*Mimics in vivo conditions* : Represents natural barrier interfaces like many mucosal surfaces. *Flexibility* : Can be applied to different cell types and tissues. *Suitable for long-term culture* : Supports differentiated and polarized cells	*Complexity* : Requires careful control of conditions and handling. *Cost* : May be more expensive due to specialized equipment	77 74
HuMiX	*Human–microbe interaction modeling* : Specifically designed to study human–microbe interactions. Strict anaerobes can be introduced. *Controlled environment* : Precise control over various factors like pH, temperature. *Integration* : Allows for integration with other methods and technologies	*Specialized requirements* : Needs unique expertise and equipment. *Cost* : Can be expensive to set up and maintain. *Limited availability* : May not be accessible to all researchers	94
Organoid-on-a-chip	*Highly realistic* : Mimics in vivo structure, function, and dynamics. *Precision control* : Allows for control over physical and biochemical conditions. *Versatility* : Can model various organs and systems	*Complexity* : Requires specialized knowledge, skills, and equipment. *High cost* : Initial setup and ongoing maintenance can be expensive. *Scalability* : May not be suitable for large-scale studies	97 113 114 115 116

**Fig.**
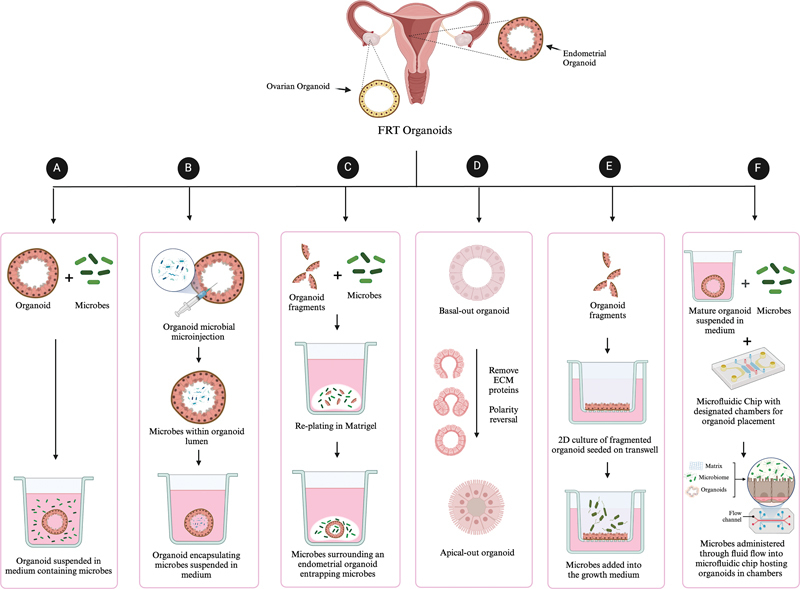
1 Integration techniques of microbes into female reproductive tract organoids. ( **a** ) *Suspension culture* : organoids are cultured in medium that allows free-floating conditions and enhances cell-to-cell interactions and nutrient absorption. Microbes are introduced into this medium, enabling interaction with the basal side of the organoid structure. ( **b** ) *Microinjection* : This technique employs a needle to inject a defined quantity of microbial suspension directly into the organoid's lumen, ensuring localized exposure of the apical side to the microbial agents and facilitating controlled studies of intraluminal microbial effects. ( **c** ) *Fragmentation* : Intact organoids are mechanically or enzymatically cleaved into smaller fragments, which are then replated and in the presence of microbial cultures both surrounding the organoid and within the organoid lumen. ( **d** ) *Apical out organoids* : The polarity of organoids is reversed to expose the apical surface, which typically lines the organoid lumen, to the external environment. This allows for the direct application of microbes onto the apical interface of the organoids. ( **e** ) *3D to 2D transition on transwell* : Organoids are dissociated to either fragments or into single cells and seeded onto a porous membrane on a transwell. The cells grow into a confluent monolayer, serving as a model of the epithelial barrier. Microbes are added to the upper chamber to study their effects across the epithelial cell layer. ( **f** ) *Organoid-on-a-chip* : This method incorporates organoids into a microfluidic system designed to emulate the physical and biochemical aspects of their native tissue environment. Organoids are cultured within defined compartments, and microbes are introduced through microchannels, allowing for real-time observation of dynamic host–microbe interactions under controlled shear stress and fluidic conditions.

### Suspension Culture

Culturing organoids in a suspension culture is a widely utilized technique in host–microbe interaction studies. 51 This approach involves the culturing of organoids in a liquid medium to which specific bacteria, isolated bacterial products, or cell-free bacteria-conditioned culture medium can be added. 35 51 52 53 54 This approach presents challenges, such as the restriction of bacterial access to the apical side of the organoid or bacterial overgrowth. Yu et al utilized this method for inoculating patient-derived FT organoids with *L. crispatus* and *Fannyhessea vaginae* . 15 Results demonstrated significant differences in the expression of inflammatory genes in organoids cultured with either bacterial species. Similarly, Koster et al utilized the suspension technique to investigate the role of pathogenic bacteria in cervical mucosa coinfections using patient-derived ectocervical organoids emphasizing the potential of patient-derived ectocervical organoids as a tool for better understanding coinfections and their role in disease development. 14

### Fragmentation

Fragmentation is a technique that involves breaking down organoids into fragments and mixing them with bacteria or bacterial products before reseeding them into the extracellular matrix gel. 10 While this method facilitates bacterial interaction with both the apical and basal facets of epithelial cells, its physiological relevance is debated. Concerns arise from inconsistencies in the quantity of bacteria or bacterial products captured within each organoid and the potential for bacteria to engage with both the basal and apical surfaces. 10 51 Despite this, two exemplary studies have used this method to study the long-term effects of *Chlamydia trachomatis* infections in the FRT. 11 12

Kessler et al utilized human FT organoids to explore the enduring effects of *C. trachomatis* infections, a leading cause of tubal infertility. 11 Long-term infected cultures consistently expanded over numerous passages and molecular analyses at 9 months revealed lasting impacts of chronic *C. trachomatis* infection, including heightened stemness, altered epithelial renewal, and DNA hypermethylation, potentially predisposing to high-grade serous ovarian cancer (HGSOC). Bishop et al fragmented murine EMOs and infected them with mCherry-expressing *C. trachomatis* . 12 This method allowed the EMOs to undergo a full developmental cycle, offering novel short-term insights into *C. trachomatis* infections. These two studies underscore the efficacy of fragmentation and pave the way for a deeper exploration of the endometrium's interactions with diverse microbes using this technique.

### Microinjection into Organoid Lumens

Microinjection is a technique that allows bacteria to contact the apical side of the epithelium while preserving the organoid's 3D structure as microbes are directly injected into organoid lumens. 55 Apical exposure is essential when studying pathogens like *Salmonella enterica* , which stimulate cytokine production in organoids only when applied to the apical side. 51 56 The injection of organoids has been extensively used in intestinal organoids and has been optimized using optimal injection volumes, fluorescent bacteria visualization, repeated microinjections, and high-throughput microinjection platforms. 55 Both commensal and pathogenic bacteria have been microinjected into organoids, with commensal bacteria maintaining species diversity for over 96 hours. 57 Repeated microinjections of genotoxic *Escherichia coli* led to mutational changes like colorectal cancer signatures, emphasizing the carcinogenic effect of this strain. 58 Microinjection offers advantages such as precise dosing of bacteria, repeated microinjections, hypoxic conditions, and longer experimental durations for studying organoid proliferation and epithelial cell subtypes. 51 58 However, it can cause structural damage to organoids, and the low oxygen in the organoid lumen makes replicating the true oxygen levels of specific FRT regions unfeasible. This approach is also susceptible to bacterial “spillage” into the basolateral compartment and is generally not ideal for high-throughput applications due to its high costs and technical challenges. 51 59 60 Despite these limitations, microinjection has proven to be a high-potential technique and is notably suited for brief analyses involving strict anaerobes due to low oxygen levels in the organoid lumen and it facilitates a direct interface between microbial and epithelial cells. 55 61 62 63

Microinjection has not been applied in many studies on the FRT. Two studies by Dolat and Valdivia utilized EMOs to investigate the interactions between *Chlamydia* and epithelial and immune cells in the upper genital tract. 13 The study revealed how *C. trachomatis* impacted the epithelial barrier and used co-cultured EMOs with mouse bone marrow–derived neutrophils to closely observe_immune cell recruitment. In subsequent research, they examined how *C. trachomatis* disrupts epithelial tight junctions by microinjecting EMO with specific bacterial strains. 64 The study revealed that *C. trachomatis* disrupts epithelial barriers by using the effector protein TepP to disassemble tight junctions early during infection. The injection of organoids offers a technical challenge, but when mastered can be especially useful when studying bacteria requiring low oxygen levels.

### Organoid Polarity Reversal

A novel technique allowing for apical interaction with bacteria, or material products, has been developed by reversing the polarity of 3D intestinal organoids by first culturing them in a basement matrix dome, then dislodging and solubilizing the domes in PBS without Ca ^2+^ or Mg ^2+^ , centrifuging the organoid suspension, and finally resuspending the pelleted organoids in growth medium in a low-attachment tissue culture plate. 65 This method ensures proper epithelial barrier integrity and nutrient uptake without the need for microinjection, letting microbes interact directly with the organoid's outward-facing apical surface. For instance, Co et al revealed that invasive pathogens such as *Salmonella* typhimurium and *Listeria monocytogenes* exhibited distinct invasion strategies for polarized epithelium, 66 while enteropathogenic *E. coli* attached primarily to the apical side of mucin-secreting cells on inverted organoids. 67

Despite these advancements, the method has notable limitations, including the time required for reversal, increased cell death, and a tendency for inverted organoids to adhere to each other in the absence of an extracellular matrix gel. 66 However, Ahmad et al were able to use this technique to study the interaction of EMOs with murine blastocysts and *E. coli* . 68 Future research is needed to understand the impact of polarity inversion on apical-out organoids' phenotype, metabolism, and microbial response, providing insights into similarities and differences between this model and self-organized organoids. Once these fundamentals have been established, more intricate investigations can be conducted. Furthermore, a shared challenge with other techniques is that there is a risk of uncontrolled bacterial growth and toxins, upsetting the equilibrium that manages bacterial proliferation and toxin neutralization. This in turn impacts organoid integrity and function, 66 increasing unpredictability in maintaining balance over extended periods, thus underscoring the importance of precision in studies centered on steady host–microbe interactions. To date, this technique has yet to be established for studying microbiome-organoid interaction with FRT organoids.

### Organoids Cultured as 2D Monolayers

Transitioning organoids into 2D monolayers offers easier access to the apical side for introducing bacteria or their by-products, overcoming challenges posed by 3D cultures such as limited uniform access, complex cell–cell interactions, heterogeneity, and restricted high-throughput application potential, all while maintaining the inherent properties of the organoid system. 69 Organoids are linearized by fragmenting 3D structures into small cell clusters or individual cells, which are then plated onto extracellular matrix-coated surfaces to form a monolayer. 70 71 72 While sacrificing the 3D structure, 2D organoid monolayers have proven their utility for studying intestinal epithelial integrity upon exposure to microbes. 73 By employing transwells to segregate the apical and basal compartments, this technique can further refine EC (endothelial cell) differentiation and allow for the inclusion of additional host factors like mesenchymal cells or immune cells, more closely mimicking the in vivo environment of the FRT. 74 Furthermore, organoids on a transwell can be cultured with medium in both the apical and basal compartments or as an air–liquid interface culture. 74 The air–liquid interphase approach provides a physiologically relevant environment for certain cell types, exposing the apical side of the monolayer to air while the basal side stays submerged in media. 75 76 Expanding on this, Zhu et al developed a 3D air–liquid interphase culture with vaginal epithelium to study herpes simplex virus-2 (HSV-2) infections. This model not only demonstrates HSV-2 susceptibility but also offers valuable insights into infection mechanisms, potential drug targets, and therapeutic efficacy evaluations. 77

However, while 2D structures on transwell plates offer easier access to the apical side, a simplified structure, and suitability for high-throughput applications, they may also lead to the loss of intricate 3D interactions, potentially misrepresent the heterogeneity and maturation of organoids, and necessitate a higher number of ECs for initiation. 75 76

### Organoid-on-a-Chip

In the last decade, advances in tissue engineering, biofabrication, and microfluidics gave rise to “organ-on-a-chip” technology. 78 It is an engineered microfluidic 3D device which mimics the microarchitecture and functions of human tissues and organs. Each chip contains a polymer that has microfluidic channels lined by living tissue-specific cells and endothelial cells, under fluid flow conditions and mechanical forces to mimic organ movements. These microdevices can combine the different cell and tissue types making up human organs, thus representing an ideal approach to study organ function on the molecular and cellular level and mimic human-specific diseases. Each setup is different, depending on the organ's physiology. The integration of organoids with these microfluidic platforms has given birth to organoids-on-a-chip. 79 80 81 Growing organoids in a tightly regulated environment of micro-engineered system may enable the development of more realistic in vitro models not achievable with organoid approaches alone, allowing for precise spatiotemporal modulation of morphogens, nutrients, physiological forces, and vascularization by co-culturing with endothelial cells for vascularization. 78 82 83 84 This setup facilitates high-throughput drug testing, continuous organoid dynamics monitoring using embedded sensors. 78 85 86 The fluid flow in these platforms resembles in vivo conditions more closely, preventing microbial overgrowth, ensuring cell health, and promoting tissue-like organization, making them an interesting tool for studying microbiota–host interactions. 87 88

The potential of organ-on-a-chip technology in elucidating microbiota–host interactions is vividly demonstrated by Mahajan et al, who developed a “vagina-on-a-chip” model. 89 This model features a microfluidic culture system replicating the human vaginal mucosa, lined by hormone-sensitive primary vaginal epithelium interfaced with underlying stromal fibroblasts. Utilizing this innovative setup, they observed that coculture with the beneficial bacterium, *L. crispatus* , led to its successful engraftment and proliferation in the chip. This coculture also maintained an acid pH, produced both D- and L-lactate, and downregulated proinflammatory cytokines. In contrast, the presence of the non-optimal bacterium, *G. vaginalis* , elevated pH levels and increased the secretion of inflammatory cytokines, culminating in epithelial cell injury. Such precise control over oxygen and pH levels, demonstrated in this study, paves the way for future microbiome–host investigations involving different FRT tissues.

## Modeling Specific Properties of the FRT

Many microbes require specific structural and functional features to interact with their host epithelium, which could be impacted if the in vitro organoid model does not recreate relevant in vivo conditions. 51 As revealed in this section, this can involve mimicking an appropriate developmental stage, the mucus layer, and proper oxygen levels.

### Modeling Oxygen Levels

Oxygen levels, which are not uniform across different bodily environments, play a crucial role in influencing microbial localization and various biological processes in the reproductive tract. 90 Low oxygen levels prevalent in reproductive tissues foster stem cell maintenance, while anaerobic or microaerophilic conditions favor the growth of specific reproductive tract microbes. 91 Conversely, changes in local oxygen levels during inflammation and disease may dramatically alter host–microbe dynamics. 92 However, replicating these conditions within organoid models is complex, as the standard oxygen concentration in organoid cultures (around 20%) contrasts sharply with the in vivo reality of less than 5% oxygen. 17 90 Creating stable and reproducible oxygen gradients across organoid cultures presents a technical challenge, but recent advances such as microfluidic organoid culture devices and oxygen-permeable scaffolds offer promising tools for maintaining controlled oxygen levels. 78 93

The human–microbial crosstalk module (HuMiX) represents a significant advancement in this field, allowing anaerobic bacteria to be maintained in an almost anoxic compartment. 94 The anoxic environment is created by perfusing an anoxic medium. Established in gastrointestinal host–microbe interaction systems, HuMiX consists of three parallel microfluidic chambers separated by semipermeable membranes with a modular design that facilitates disassembly and cell collection. Successfully co-cultured with anaerobic bacteria like *Bacteroides caccae* and *Lactobacillus rhamnosus* , as well as immune cells, the system is being further developed to become the immuno-HuMiX, allowing the coexistence of patient-derived microbiota, ECs, and immune cells, although the mucus-coated membrane currently prevents direct host–microbe contact. 94 Defining the optimal oxygen conditions for organoids and associated microbes is significant when developing a host–microbiome in vitro model.

### Modeling Age-Specific Properties of the FRT Epithelium

Modeling age-specific properties of the FRT epithelium is a key component for understanding fertility, particularly as aging profoundly affects the FRT's physiology. 95 EMOs derived from postmenopausal women exhibit altered morphology and reduced hormone responsiveness, highlighting the utility of organoids in modeling age-related properties of the FRT epithelium. 38 This also means that when generating adult stem cell-derived organoids, features like the age of the donor need to be accounted for, especially when conducting host–microbe studies, as age-specific properties have been shown to affect microbial communities. For instance, the ovulatory cycle's influence on microbial composition, with estrogen and progesterone causing changes in epithelial thickness and glycogen deposition, leads to different community state types with various *Lactobacillus* species. 91 Menopause results in a reduction in *Lactobacilli* , associated with higher follicle-stimulating hormone (FSH) levels and lower estrogen levels, linked to vaginal dryness and atrophy. 91 Moreover, postmenopausal estrogen deficit affects the vaginal microbiome, reducing *Lactobacilli* and correlating with decreased serum estrogen levels. 91 These insights into the age-specific properties of the FRT epithelium and their relationship with microbial interactions are vital for fertility studies. The ability to model these complex dynamics through organoids and other methods offers promising avenues for understanding and potentially addressing fertility challenges across various stages of a woman's life.

### Modeling the Influences of the Hormonal Cycle

The FRT is a dynamic system where the endometrium, ovaries, and cervix are influenced by hormonal changes that occur throughout the menstrual cycle, affecting cellular and molecular events, resident microbiota, and thereby host–microbe interactions. 95 These hormonal shifts, like rising estrogen levels, induce remarkable remodeling in the endometrium, affecting aspects like the proliferation of glands and the maturation of functional layers. 96 Turco et al used organoids to model how glandular EMOs respond to ovarian sex hormones, revealing specific hormonal effects on differentiation and showing the potential for studying the FRT microbiota's role in these processes. 38 Additionally, a microfluidic ovary–FT coculture device has demonstrated estrogen-mediated interorgan signaling. 97 Modeling the hormonal cycle in vitro is vital, as it would enable the study of dynamic changes such as pH, nutrient availability, and immune factors that influence host–microbe interactions throughout the cycle.

### Modeling the Mucus Layer

The mucus layer in the FRT is vital for reproductive functions, with its properties varying due to hormonal changes, ensuring a conducive environment for fertilization and embryo gestation. 98 The cervico-vaginal tract acts as a barrier, balancing sperm passage, supporting beneficial bacteria, and warding off harmful pathogens. 99 100 Disturbances, like bacterial vaginosis, can cause health issues such as preterm birth and increased HIV-1 risk. 101 102 Hormonal cycles, especially estrogen and progesterone, influence mucus consistency and acidity. 103 While healthy mucus promotes *Lactobacillus* adherence and restricts pathogens, dysbiosis can impair fertility and lead to diseases like endometritis. 104 105 106 There is a need for human in vitro models that accurately represent mucus composition, structure, and function. Research has utilized various in vitro models, such as conventional cultures, transwell inserts, 3D-engineered constructs, and organoid models, to tackle these challenges. However, these models have limitations, and human organ-on-a-chip technology has been applied to overcome these challenges and model mucus biology more physiologically. 98 One notable advancement was made by Izadifar et al, who developed a two-channel cervical chip lined with primary human cervical ECs. 18 This innovation, along with the human vaginal chip, has provided insights into mucus physiology and pathophysiology. 89 A simplified alternative, the mucus chip, has also emerged, offering insights into mucus penetration for drug delivery. 107 Organ-on-a-chip technology's potential extends beyond modeling, facilitating simultaneous characterization of mucus biochemical, structural, and biophysical properties. 98 For future research, it would be useful to see if such FRT organoids-on-a-chip models can be made using organoid-derived cells or linearized organoids to have an accessible mucus layer. Then the properties between the differently constructed models' mucus layers could be assessed, both with microbial cultures and without. In summation, human organ-on-a-chip models represent an innovative platform for conducting mechanistic studies of mucus formation, functions, and underlying influences of tissue biology, holding great promise for studies on host–microbe interactions in the FRT.

### Integrating Components of the Immune System

The mucus layer in the FRT regulates the microbial community, serving as a barrier that influences immune responses and reproductive health through intricate interactions. 108 109 110 111 Imbalances in reproductive tract microorganisms, metabolites, or immunity can disrupt this harmony, potentially leading to disease development. 112 Therefore, incorporating immune cells into organoid models enhances their ability to model infection, inflammation, and other disease processes driven by immune–microbe interactions. Incorporating immune cells into organoid structures entails methods like direct luminal injection, addition to culture medium, or leveraging organoids with inherent immune cells, the latter highlighted by Yu et al. 15 However, achieving uniform incorporation, phenotype maintenance, and accounting for variability in immune cell sources present challenges.

## Conclusion

Organoid-derived technologies have significantly advanced the study of the FRT, filling the gap between traditional cell cultures and animal models. These advancements provide a basis for further refinement and standardization of these models. A defined research direction is crucial. Efforts should focus on standardizing FRT organoid–microbe co-cultures, considering variables such as donor age, hormonal cycles, and disease conditions. By addressing these elements, the creation of advanced models that simulate both aerobic and anaerobic environments becomes more achievable, leading to a closer representation of in vivo conditions suitable for both short- and long-term co-cultures. The subsequent step involves the development of interconnected multi-organoid systems, enhanced with microfluidic technologies, to reflect the cellular dynamics of the FRT more accurately. These models should aim to include vascularization, immune components, stromal elements, and other essential cell types. However, technical challenges, such as determining optimal flow rates and formulating compatible media, demand concentrated research efforts to ensure interactions with diverse cell types, microbes, and hormones.

Investigating conditions like endometriosis using patient-specific organoid models is of paramount importance. Additionally, the merging of organoid technology with genomics, proteomics, and interdisciplinary research offers a broader perspective on FRT health. 40 Improvements in co-culture systems, biomaterials, and integrated screenings are promising pathways toward mimicking the in vivo microenvironment more closely. In conclusion, as organoid-based models continue to progress, their combination with microfluidic technologies, despite inherent challenges, stands to enhance our understanding of microbial roles in reproductive health, setting the stage for tailored therapeutic approaches in reproductive healthcare.
